# Isolation, Structures, and Bioactivities of the Polysaccharides from* Gynostemma pentaphyllum* (Thunb.) Makino: A Review

**DOI:** 10.1155/2018/6285134

**Published:** 2018-10-16

**Authors:** Xiaolong Ji, Yingbin Shen, Xudan Guo

**Affiliations:** ^1^College of Food Science and Engineering, Northwest A&F University, 712100 Yangling, China; ^2^Department of Food Science and Engineering, Jinan University, Guangzhou 510632, Guangdong, China; ^3^Basic Medical College, Hebei University of Chinese Medicine, 050200 Shijiazhuang, China

## Abstract

Polysaccharides obtained from* Gynostemma pentaphyllum* (Thunb.) Makino have promising prospects in functional food and nutraceuticals due to its broad range of biological activities including antioxidant, immunomodulatory, antitumor, hepatoprotective, neuroprotective, and antifatigue activities. These beneficial biological activities are related to chemical composition and structure of the* G. pentaphyllum *polysaccharides. The molecular weight, monosaccharide composition, and chemical structures could be influenced by both different extraction/purification techniques employed to obtain polysaccharide enriched products. The purpose of this article is to review previous and current literature regarding the extraction, purification, structural characterization, and biological activity of* G. pentaphyllum* polysaccharides. This review provides a useful bibliography for the further investigation, production, and application of* G. pentaphyllum* polysaccharides as functional foods and nutraceuticals.

## 1. Introduction

*Gynostemma pentaphyllum* (Thunb.) Makino, named “Jiao-Gu-Lan” in Chinese, belongs to the family* Cucurbitaceae *and genus* Gynostemma Bl.* and is distributed widely in northeast and southeast Asia [1–3].* G. pentaphyllum* has been used in food and supplemental products for hundreds of years in China, where it is mainly distributed south of the Qinling Mountains and Yangtze River [4, 5]. According to the traditional Chinese medicine, the taste and nature of* G. pentaphyllum* are slightly bitter, neutral, and warm [6].* G. pentaphyllum* consumption is believed to treat hematuria, edema, pain of the pharynx, heat and edema of the neck, and tumors and trauma [7]. The book, Herbs for Famine, published during the Ming Dynasty (1368-1644 AD) described the use of* G. pentaphyllum* as a vegetable, which was suitable as a food or a dietary supplement during famine [6, 8]. At present, lots of* G. pentaphyllum* products have been launched in the United States, China, and several other Asian and European countries, including* G. pentaphyllum* tea, tablet, instant powder, capsule, oral liquid, and pill. In addition, there are additives made from* G. pentaphyllum* for use in beverage, sports drink, cola, beer, biscuits, breads, and noodles [9, 10].

In recent decades, pharmacological studies have reported many functions of* G. pentaphyllum*, including antimicrobial, anticancer, antiaging, antifatigue, antiulcer, hypolipidemic, and immune-modulatory activities [11–15]. The multiple pharmacological effects of* G. pentaphyllum* are attributed to its various chemical ingredients, including saponins, amino acids, polysaccharides, flavonoids, organic acids, trace elements, and other chemicals [16, 17]. Polysaccharides are one of the most abundant components of* G. pentaphyllum* and represent a major group of biologically active constituents.* G. pentaphyllum* polysaccharides isolated with different extraction and purification methods have been shown to be structurally diverse biomacromolecules with various functions, including anti-inflammatory [18], antitumor [19], immunomodulatory [20, 21], and antioxidant activities [22], antiexercise fatigue properties [23, 24], hepatoprotective [25], and neuroprotective [26] activities and as a therapeutic agent for the treatment hyperlipidemia disorders [27].

To the best of our knowledge, there has been no review of the extraction and purification techniques or the structural characteristics and biological activities of* G. pentaphyllum* polysaccharides. One of the purposes of this review is therefore to report the relationships between the structural features and biological activities of* G. pentaphyllum* polysaccharides in order, to aid in the better understanding and subsequent utilization of these macromolecules.

## 2. Extraction and Purification Methods

**Figure 1:**
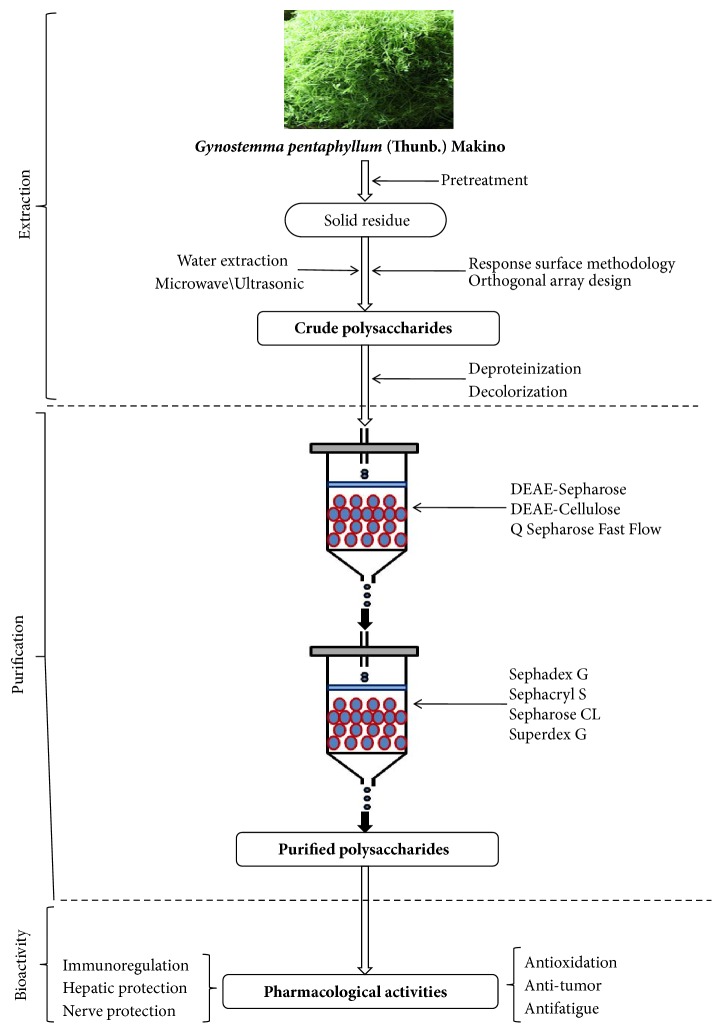
Schematic representation of the extraction, purification, and bioactivity of polysaccharides from the* Gynostemma pentaphyllum*.

As* G. pentaphyllum *polysaccharides are structural components of cell walls, basic extraction methods are used that breakdown the cell wall from the outer layer to the inner layer with mild to strong extraction conditions, which do not alter the structural morphology of the cell well [28–32]. The list of the extraction methods for* G. pentaphyllum* polysaccharides obtained from pretreated dry powders is summarized in Table 1. Generally, extraction in hot or boiling water is the classical and most convenient method of laboratory extraction and is also widely used in industry [30, 31]. The liquid: solid ratio has an important influence on the yield for conventional water extraction, and the extraction temperature together with time is usually in the range of 80-100°C and 15-360 min, respectively. However, the disadvantages of hot water extraction include long times and high temperatures, low efficiency, and possible polysaccharides degradation [33]. Different technologies have been used to improve the efficiency of extraction, including microwave-assisted treatments, high-powered ultrasonic processing, and enzyme assistant extraction. Response surface methodology based on a Box-Behnken design or Central Composite Rotatable design was applied to optimize extraction conditions to obtain the crude* G. pentaphyllum *polysaccharides through water extraction and ethanol precipitation [34–37]. Taken together, with the application of various technologies, a higher yield could be obtained, even with fewer and shorter extraction times, with lower extraction temperatures, and with smaller solid: liquid ratios [28].  Figure 1 illustrates the extraction and purification of* G. pentaphyllum* polysaccharides. An ultrasonic and microwave-assisted extraction method is used to maximize the output of* G. pentaphyllum* polysaccharides. The most favorable conditions for this extraction originated in the Qinling Mountains of Shaanxi Province and include ultrasonic power of 900 W, an extraction time of 40 min, and a liquid: solid ratio of 1:25, to produce a final yield of 7.29% [38]. Zhou identified the optimal extraction conditions as a microwave power of 800 W, microwave time of 15 min, and a liquid: solid ratio of 1:35. Under these conditions, the yield of crude polysaccharides from* G. pentaphyllum* was 8.61% [38].

Crude* G. pentaphyllum* polysaccharides can be further purified by a combination of techniques, including precipitation with ethanol, protein removal by the Savage reagent, decolorization by H_2_O_2_ or macroporous resin, ion exchange chromatography, and gel filtration chromatography [39, 40]. Ion exchange chromatography separates neutral polysaccharides from acidic ones using various concentrations of an NaCl eluent. Gel filtration separates polysaccharides of different molecular weights. Li et al. isolated three different fractions (GPA1, GPA2, and GPA3) from acidic* G. pentaphyllum* polysaccharides separated with a diethylaminoethyl-cellulose column (7.0 × 30 cm) and a Sepharose CL-6B column (2.5 × 100 cm). These three acidic polysaccharides contained different amounts of Man, Rha, GlcA, GalA, Glc, Gal, Xyl, Ara, and Fuc [4]. Jia et al. fractionated GPP1, GPP2, and GPP3 with a DEAE cellulose column (2.0 × 40 cm) preequilibrated with distilled water and eluted with 0, 0.3, and 2.5 M of NaCl at a flow rate of 1 mL/min (10 mL/tube). The collected fraction was further purified on a Sephacryl S-400 column (3.0 × 100 cm) and eluted with distilled water at a flow rate of 0.3 mL/min. The major polysaccharide fraction was collected and freeze-dried to give a white purified polysaccharide [41].

The procedures used to separate and purify the polysaccharides from* G. pentaphyllum* are summarized as follows. Briefly,* G. pentaphyllum* is carefully washed, dried, and ground to obtain a fine powder and then immersed in 80% ethanol for hours to remove fat, pigments, and low molecular weight sugars. The polysaccharide solution is then extracted from the residue with water using differentially assisted extraction steps and is then filtered and concentrated [42, 43]. After solubilization, the resultant polysaccharide solution is usually subjected to different chromatographic columns described above and sequentially eluted with appropriate running buffers, collected, dialyzed, concentrated, and lyophilized, to produce the pure* G. pentaphyllum* polysaccharides [41, 42]. The polysaccharide contents can be determined using the phenol-sulfuric acid method [44]. The polysaccharides isolated from* G. pentaphyllum *are used to make oral liquid, sports drink and chewable tablet.

## 3. Physiochemical and Structural Features

The physiochemical and structural characteristics of a polysaccharide mainly include monosaccharide composition and sequence, molecular weight, configurations, types, and positions of glycosidic linkages [45–47]. Polysaccharides with various monosaccharide constituents and chemical structures have been isolated from* G. pentaphyllum*. Different research groups determined the basic chemical structures of purified* G. pentaphyllum* polysaccharides using gas chromatography, gas chromatography-mass spectroscopy, infrared spectroscopy, nuclear magnetic resonance (NMR), high-performance liquid chromatography (HPLC), acid hydrolysis, methylation analysis, periodate oxidation, and Smith degradation [15, 22]. The primary structural characteristics of* G. pentaphyllum *polysaccharides, such as their molecular weights, monosaccharide compositions, chemical structures, and biological activities, are summarized in Table 2, together with their names and related bibliographies.

### 3.1. Monosaccharide Compositions

Monosaccharide composition analyses commonly involve the cleavage of glycosidic linkages by acid hydrolysis, derivatization, and detection and quantification with HPLC and gas chromatography methods [30, 48]. Because different raw materials, extractions, and purification processes have been used, different monosaccharide compositions of* G. pentaphyllum* polysaccharides have been reported, but most of the polysaccharides are composed of Rha, Man, Ara, Glc, and Gal in different molar ratios. Li et al. separated three polysaccharides, GPA1, GPA2, and GPA3, from* G. pentaphyllum* and analyzed their monosaccharide compositions with HPLC [4]. The results are shown in Table 2. Song and colleagues reported the monosaccharide compositions of two polysaccharides (GPS-2 and GPS-3) and found that GPS-3 consisted of Rha, Xyl, Ara, Gal, and Glc in a molar ratio of 1.75:1.00:8.70:3.07:5.79, whereas GPS-2 consisted only of Rha and Xyl [49, 50]. Various* G. pentaphyllum* polysaccharides have different monosaccharide compositions in various molar ratios. Indeed, the same variety* G. pentaphyllum* in different fields may have different monosaccharide compositions.

### 3.2. Average Molecular Weights

Different techniques including HPLC and high-performance gel permeation chromatography have been used to determine the average molecular weights of* G. pentaphyllum* polysaccharides, with many studies of* G. pentaphyllum* polysaccharides based on the same methods [42, 43]. Chi et al. reported that the molecular weights of* G. pentaphyllum* polysaccharides were 8.920 × 10^4^ Da (GPP1-a), 1.975 × 10^5^ Da (GPP2-b), and 2.536×10^5^ Da (GPP3-a) [22]. Different molecular weights in the range of 10^3^-10^6^ Da have been found in various* G. pentaphyllum* preparations using different experimental conditions.

### 3.3. Chemical Structures

**Figure 2:**
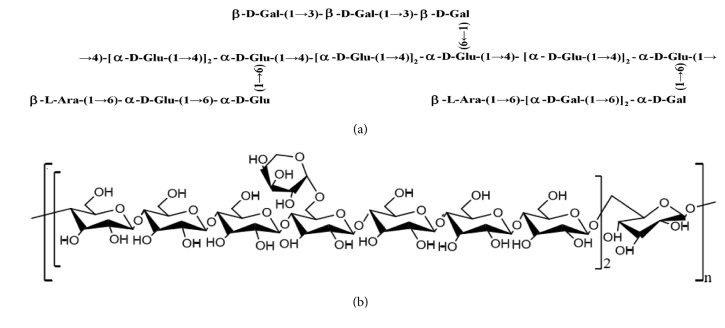
Schematic structure of GPP1-a (a) and GPP-S (b).

Apart from their monosaccharide components and molecular weights, little structural or conformational information regarding* G. pentaphyllum* polysaccharides has been reported. A structural investigation of a* G. pentaphyllum* polysaccharide with antiexercise fatigue activity (GPP1-a) indicated that GPP1-a (Figure 2(a)) contained a backbone of (1→4)-linked *α*-D-glucose residues, with branches attached at O-6. The branches were mainly composed of (1→6)-linked *α*-d-glucose, (1→3)-linked *β*-D-galactose, and (1→6)-linked *α*-d-galactose residues and terminated with *β*-_L_-arabinose residues [22].

The structural features of a water-soluble polysaccharide (GPP-S) were studied using methylation analysis, Fourier transform-infrared spectroscopy, and ^1^H, ^13^C, and HSQC, COSY, and HMBC NMR spectral data. It was shown that the GPP-S primarily consisted of (1→4)-linked-Glc*p* (76.37%), (1→4,6)-linked-Glc*p* (12.42%), (1→6)-linked-Gal*p *(6.74%), and (1→)-linked-Ara*f *(4.47%); a schematic structure is shown in Figure 2(b) [18].

The primary structures of* G. pentaphyllum* polysaccharide (GPP-TL) were determined with a combination of chemical and instrumental analyses, including methylation analysis, gas chromatography, infrared spectroscopy, and ^1^H and ^13^C NMR. GPP-TL had glucose and galactose residues in the main chain with (1→6)-linked branches at glucose residues [9].

### 3.4. Conformational Features

Polysaccharide activities depend on their chemical structures, molecular weights, and chain conformations, but no reports are available on the chain conformations of* G. pentaphyllum* polysaccharides [30, 51]. Except for a study by Chi et al., no reports have described scanning electron microscopic and atomic force microscopic structural characterization of GPP1-a. GPP1-a consisted mainly of randomly distributed individual spherical particles, which were comprised of smaller spherical particles with diameters of 500-1000 nm. There were many clusters with different sizes, which were attributed to the aggregation of one or more GPP 1-a polysaccharide chains at room temperature [22].

The relationships between the chain conformations of* G. pentaphyllum* polysaccharides and their biological activities are difficult to determine [46, 48]. The details of the chain conformations of* G. pentaphyllum* polysaccharides in aqueous solution require further investigation with advanced technologies, such as viscosity analyses, static and dynamic light scattering, circular dichroism, transmission and scanning electron microscopy, atomic force microscopy, fluorescence spectroscopy, and NMR spectroscopy [48, 52].

## 4. Biological Activities

Based on traditional Chinese medicine theory,* G. pentaphyllum* is widely used to reduce cholesterol levels, promote the production of body fluids, regulate blood pressure, strengthen the immune system, treat chronic bronchitis and gastritis, and reduce inflammation [63–66]. According to many studies, polysaccharides are a major class of bioactive compounds in* G. pentaphyllum*, contributing to its beneficial effects on human health and its pharmacological activities. The multiple bioactivities and health benefits of* G. pentaphyllum* polysaccharides are summarized and compared in detail below.

### 4.1. Antioxidant Activity

Natural materials are a highly promising source of antioxidants, and a wide range of bioactive constituents of plants, fungi, and animals, especially polysaccharides, have antioxidant activities [67–70]. Antioxidant activities have been the focus of much research into the mechanisms underlying the nutraceutical and therapeutic effects of traditional Chinese medicines, based on various assay methods and activity indices [46, 67].

Many research groups have demonstrated the antioxidant activities of* G. pentaphyllum* polysaccharides* in vitro *and* in vivo*. Li et al. recently demonstrated that GPA1, GPA2, and GPA3, obtained from* G. pentaphyllum* using a combination of water extraction, ion exchange, and gel permeation chromatography had antioxidant activities [4]. Results showed that GPA3 had a stronger scavenging activity of 2,2-diphenyl-1-picrylhydrazyl (DPPH) and hydroxyl radicals; a stronger chelating activity of ferrous ions; and a stronger reducing power than GPA1 and GPA2* in vitro*. A novel heteropolysaccharide (GPP-TL) isolated from tetraploid* G. pentaphyllum *(Makino) leaf by hot water extraction, anion exchange, and gel permeation chromatography had a DPPH^•^ scavenging capacity value of 15.92 *μ*mol TE/g; a HOSC value of 36.42 *μ*mol TE/g; and an ORAC value of 10.83 *μ*mol TE/g under the experimental conditions [9]. Three fractions of polysaccharides, GMA, GMB, and GMC, were isolated and purified from* G. pentaphyllum*, and their antioxidant activities were evaluated using superoxide radical, hydroxyl radical, and 1,2,3-phentriol self-oxidation assays [62]. The results indicated that GMC possessed a strong scavenging effect of superoxide radicals and inhibited 1,2,3-phentriol self-oxidation, which may have been associated with the physiochemical and monosaccharide composition of these polysaccharides.

GPP1-a, composed of Ara, Gal, and Glc with molar ratios of 0.18:0.72:1.00, significantly prolonged the exercise time to exhaustion in mice; increased glycogen levels and some of antioxidant enzyme activities; and decreased malondialdehyde (MDA) levels in muscle. The results showing that GPP1-a prolonged exercise time to exhaustion in mice may have been associated with scavenging of reactive oxygen species (ROS) [22]. The antioxidant activities of* G. pentaphyllum* polysaccharides* in vivo* were reported to be less definitive than* in vitro* results, indicating that mechanism of polysaccharide antioxidation needs further exploration.

### 4.2. Immunomodulatory Activity

Immunomodulation is considered an important biological function of natural polysaccharides, which act as immunomodulators and/or biological response modifiers [71, 72]. Studies found that* G. pentaphyllum* polysaccharides promoted cellular immunity, humoral immunity, and nonspecific immunity. The immunomodulatory activities of* G. pentaphyllum* polysaccharide conjugates (GPMPP) were previously investigated by Shang et al. in rats [13]. Their results indicated that GPMPP significantly increased splenic and thymic indices; activated macrophages and NK cells; and exhibited activity on normal and Con A/LPS-stimulated splenocytes in a dose-dependent manner in C57BL/6 rats. GPMPP elevated CD4^+^ T lymphocyte counts as well as the CD4^+^/CD8^+^ ratio in a dose-dependent manner and it increased IL-2 levels in the sera and spleen of Cy-immunosuppressed mice. Furthermore, GPMPP also significantly increased SOD, GSH-Px, T-AOC, GSH, and CAT levels and decreased MDA levels. The results showed that GPMPP might play an important role in prevention of oxidative damage in the immune system and indicated that GPMPP had immunomodulatory activity* in vivo*. Yang et al. reported that PSGP (a water-soluble polysaccharide from* G. pentaphyllum* herbal tea) reduced peritoneal macrophages to release nitric oxide, ROS, and tumor necrosis factor-alpha and inhibited the proliferation of human colon carcinoma HT-29 and SW-116 cells* in vitro* in a dose-dependent manner [73].

### 4.3. Antitumor Activity

It has been reported that the anticancer effects of polysaccharides have strong relationship with their molecules size form, degree of branching, and solubility in water. As we have mentioned before, many previously studies have suggested that polysaccharides exert strong antitumor activity through different mechanisms [30, 74, 75], (1) the prevention of oncogenesis by oral administration of polysaccharides; (2) improving the immune response to tumors; (3) direct antitumor activity through inducing the apoptosis of tumor cells; and (4) preventing the spread or migration of tumor cells in the body [30, 72–76]. Li et al. reported that several* G. pentaphyllum* polysaccharide fractions (GP-B1 and GP-C1) had a significant inhibitory effect on the growth of melanoma B16 cells* in vivo* and* in vitro* [42]. However, GP-B1 and GP-C1 are dissimilar in their chemical compositions and molecular weights, and the lower molecular weight form of GP-B1 had higher antitumor activities. The antitumor actions of acidic polysaccharides were associated with their molecular weights, chemical compositions, and glycosidic linkages [30].

### 4.4. Hepatoprotective Activity

Only a few studies have demonstrated the direct hepatoprotective effects of* G. pentaphyllum* polysaccharides. Therefore, more detailed studies are required to clarify the compositional features and hepatoprotective activities of these polysaccharides. Song et al. reported that administration of GPS-3 at doses of 50, 100, or 200 mg/kg body weight prevented the hepatic injury induced by erguotou liquor (16 mL/kg) in mice in a dose-dependent manner. Furthermore, at these dosages, GPS-3 could significantly inhibit increases in serum AST and ALT levels, reduce hepatocyte MDA content, increase GSH content, and reduce hepatocyte necrosis in the injured mice [50].

Low and high doses of* G. pentaphyllum* polysaccharides (40 and 80 g/kg, respectively) were fed to rats with injured livers induced by carbon tetrachloride.* G. pentaphyllum* polysaccharides significantly decreased the levels of AST and ALT in liver-injured rats, while iNOS mRNA expression in hepatic tissue was downregulated. In addition, levels of the antiapoptotic protein, Bcl-2/Bax, were elevated in hepatic tissue and there was reduced liver injury. The results indicated that* G. pentaphyllum* polysaccharides had protective effects on CCl_4_-induced liver injury in rats, and whose mechanism of action may have been related to the inhibition of cytotoxicity and antiapoptotic pathways [77].

### 4.5. Neuroprotective Activity

Many research groups have investigated the neuroprotective effects of polysaccharides in different cell models [78–80].* In vivo *and* in vitro *studies have demonstrated the ability of polysaccharide-rich extracts to provide neuroprotective effects through promotion of neurite outgrowth and activation of NF-*κ*B, PI3K/Akt, MAPK, Nrf2/HO-1 signaling pathways [81]. GPP1 (a purified polysaccharide from* G. pentaphyllum*) efficiently protected PC-12 cells against A*β* (25-35)-induced cytotoxicity, likely by either preventing oxidative stress, excessive intracellular free calcium concentration influx, or loss of mitochondrial membrane potential or through elevating Bax/Bcl-2 and cleaved caspase-3 protein expression or possibly by a combination of these effects. These findings suggested that GPP1 exerted a neuroprotective effect against A*β* (25-35)-induced neurotoxicity in PC12 cells, at least in part, via inhibiting oxidative stress and suppressing the mitochondrial apoptotic pathway [41].

### 4.6. Antifatigue Activity

The consumption and depletion of energy sources [82], the production and accumulation of metabolic products [83], the dysfunction of the immune system [84], and excessive generation of ROS, which are highly reactive molecules that can attack and damage cellular structure, all promote exercise-induced fatigue [85, 86]. Many studies have attempted to identify natural antifatigue polysaccharides without adverse effects, to improve athletic ability, postpone fatigue, and to accelerate the elimination of fatigue in humans [87]. Treatment with GPP1-a significantly prolonged exhaustive exercise time of mice. The underlying mechanisms by which GPP1-a prolonged this exhaustive exercise time may have been associated with the role of GPP1-a in scavenging excessive ROS produced during the exercise regime [60].

### 4.7. Others

*G. pentaphyllum* polysaccharides were shown to have significant* in vivo* antidiabetic effects in a type 2 diabetes rat model induced by injection of streptozotocin after consumption of a high fat/sugar diet. Polysaccharide administration significantly lowered levels of blood glucose levels, total cholesterol, triglycerides, low-density lipoprotein, and malondialdehyde and increased blood insulin, superoxide dismutase, and high-density lipoprotein. The results indicated that* G. pentaphyllum* polysaccharides had hypoglycemic and hypolipidemic effects in rats with streptozotocin-induced type 2 diabetes and that the underlying mechanism associated with these effects might have been related to increases in serum insulin and antioxidant activity [27].

Pharmacological studies of polysaccharides have shed some light on a novel aspect of functional foods in antiaging [88, 89]. The most obvious was the 55.44% inhibition of COX-2 by GPP-S. The inhibition of IL-1*β* and IL-6 was 30.58% and 20.54%, respectively [18].

## 5. Correlations of Structure, Content, and Biological Activity

The various biological activities of polysaccharides are strongly related to their chemical compositions and configurations [39, 45]. Few studies regarding the structure-function relationships of these polysaccharides have been reported, and it has been difficult to relate the structures of* G. pentaphyllum* polysaccharides to their biological activities. Nevertheless, some relationships can be inferred as follows.

It is well-established that the molecular weights of polysaccharides are closely associated with their biological activities [90, 91]. Li et al. prepared a lower molecular weight polysaccharide (GPA3) with a similar composition to other polysaccharides (GPA1 and GPA2), which displayed higher antioxidant activities than GPA1 and GPA2 because its lower molecular weight allowed the spatial conformation of* G. pentaphyllum *polysaccharides to be maintained [4]. Antioxidation tests* in vitro* showed that GMC (72 kDa) possessed a stronger scavenging effect of superoxide radicals and inhibited the activity of 1,2,3-phentriol self-oxidation more than GMA (94 kDa) and GMB (120 kDa) [62].

Many glycoconjugates are acidic complex carbohydrates composed of glucuronic acid and galacturonic acid [92]. Uronic acid residues can alter the physiochemical properties and solubility of the associated polysaccharide conjugates and therefore can affect the activities of polysaccharides [31, 61]. Uronic acid in* G. pentaphyllum* polysaccharides is crucial for their biological activities, and fractions rich in uronic acid have higher bioactivity. GP-C1 contains a similar monosaccharide composition as GP-I and has greater antitumor activities than GP-I* in vitro*, most likely do the fact that GP-C1 contains galacturonic acids [42, 43].

Previous studies have indicated that the structural characteristics of polysaccharides, such as *α*-(1→4) linkages in the main chain, are important for their biological activities [39]. However, various chemical structures have been reported for* G. pentaphyllum* polysaccharides including a backbone composed of (1→4)-*α*-d-Glc*p* (Table 2). Overall, different studies have expedited our understanding of the structural basis of the biological effects and biological mechanisms of polysaccharides.

## 6. Conclusions and Perspectives

*G. pentaphyllum* (Thunb.) Makino is a source of highly promising traditional medicines and functional foods and has thus gained increasing attention. Over the past thirty years, polysaccharides have been isolated and purified from* G. pentaphyllum* with various extraction methods, mainly microwave-assisted or ultrasonic-assisted.* G. pentaphyllum* polysaccharides have a wide range of potent bioactivities, including antioxidant, immunomodulatory, antitumor, hepatoprotective, neuroprotective, and antifatigue activities. Like many other polysaccharides [30], the isolation, structural characterization, and bioactivities of polysaccharides from* G. pentaphyllum* have been extensively investigated in recent years. However, the relationships between their bioactivities and these high-order structural chemicals are still not well-established because of the great diversity and complexity of the latter. Further research is required to extend our understanding of the functional effects of* G. pentaphyllum* polysaccharides.

To better determine the effects of* G. pentaphyllum* polysaccharide metabolites on human health,* in vivo* studies must be conducted, both in animals and clinical studies, because the limitation of those* in vitro* studies carried on human tissues and cells. Another important issue is the exploration of potent new technologies, such as the “omics” technologies (i.e., genomics, transcriptomics, metabolomics, and proteomics) and bioinformatics to clarify the different mechanisms underlying the effects of* G. pentaphyllum* polysaccharides on their bioactivities. This knowledge will help investigators to design more potent health promoting pharmaceuticals and functional foods based on* G. pentaphyllum* polysaccharide chemical modifications.

## Figures and Tables

**Table 1:** A summary of the extraction of polysaccharides from *Gynostemma pentaphyllum*.

Types	Times (min)	Solid-liquid ratio	Temperature (°C)	Solvent	Other conditions	Yield (%)	References
*Routine extraction*							
GPMPP	90	1:15	95	water	4 times		[36]
CGP	15	1:67	95	water	immersing time: 10 min	11.29	[37]
CGP	30	1:10	100	water		6.82	[49]
CGP	120	1:20	100	water	2 times	6.35	[38]
CGP	120	1:15	80	water		2.82	[34]
CGP	180	1:40	80	water	2 times	5.35	[53]
CGP	120	1:40	85	water	2 times		[54]
CGP	120	1:15	90	water	2 times	4.03	[55]
CGP	60	1:16	80	water		9.66	[56]
GPP	120	1:15	80	water	2 times	11.44	[22]
CGP	120	1:16	80	alkali solution	0.5 M NaOH		[18]
CGP	360	1:16		alkali solution	0.5 M NaOH		[56]
*Ultrasound-assisted extraction*							
CGP	40	1:25		water	ultrasonic power 900W 2 times	7.29	[38]
CGP	31	1:26	83	water		3.356	[57]
CGP	15		50	water	ultrasonic power 800W	2.49	[35]
GPP	52	1:47	100	water	ultrasonic power 129W	3.24	[58]
*Microwave-assisted extraction*							
CGP	15	1:35		water	microwave power 800W 2 times	8.61	[38]
CGP	6	1:25			microwave power 560W immersing time: 70 min	3.91	[55]
GGP	12	1:20			microwave power 400W immersing time: 50 min	3.37	[34]
*Enzyme-assisted extraction*							
CGP	150		45	water	ratio of enzyme amount 2%, pH 6.0		[59]

**Table 2:** The polysaccharides isolated from *Gynostemma pentaphyllum*.

No.	Compound name	Molecular weight (Da)	Monosaccharide composition	Structures	Biological activities	Reference
1	GPMPP	3.67×10^4^	Rha, Ara, Xyl, Man, Glc, Gal in the ratio of 1.39:3.76:1.00:1.64:4.98: 5.88		Antioxidant Immunomodulation	[13]

2	GPA1	1.96×10^4^	Man, Rha, GlcA, GalA, Glc, Gal, Ara, Fuc in the ratio of 1:0.04:1.4: 0.9:1.3:2.6:2:0.2		Antioxidant	[4]
	GPA2	1.06×10^4^	Man, Rha, GlcA, GalA, Glc, Gal, Xyl, Ara, Fuc in the ratio of 1:0.1: 2.2:1.8:2.2:2.6:0.2:1.9:0.2			
	GPA3	6.7×10^3^	Man, Rha, GlcA, GalA, Glc, Gal, Xyl, Ara, Fuc in the ratio of 1:0.6: 3.9:0.5:5.5:2.6:0.5:1.2:0.2			

3	GPP1		Glc, Man, Gal, Rha, Ara in the ratio of10.0:9.9:5.1:2.5:2.4	Backbone composed of (1→6)-linked- Glc*p*, (1→6)-linked-Man*p*, (1→3,6)- linked-Gal*p*, with braches attached to O-3 of some residues. Braches composed of (1→)-linked-Rha*p* residues and (1→)-linked-Ara*f* residues	Neuroprotective effect	[41]

4	CGPP		Man, Glc, Ara, Rha, Gal, GlcA in the ratio of 2.0:2.2:1.3:2.2:1.2:2.5		Anticancer Immunomodulation	[20]

5	GPP-TL	9.3×10^3^	Glc, Gal, Ara in the ratio of 43:5:1	Backbone composed of (1→4)-*α*-_D_-Glc*p*, (1→4)-*β*-_D_-Gal*p*, (1→4,6)-*α*-_D_-Glc*p*, with braches attached to O-6 of some residues. Braches composed of (1→)-*α*-_D_-Glc*p*	Antioxidant	[9]

6	GPS-3	9.1×10^3^	Rha, Xyl, Ara, Gal, Glc in the ratio of 1.75:1.00:8.70:3.07:5.79	*α*-configuration and *β*-configuration	Hepatoprotective activity Antitumor	[49, 50]
	GPS-2	1.07×10^4^	Rha, Xyl in the ratio of 1:12.25	*α*-configuration		

7	GPP1-a		Ara, Gal, Glc in the ratio of 0.18: 0.72:1.00	*β*-configuration	Antioxidant	[60]
	GPP2-b		Ara, Rib, Xyl, Gal, Glc in the ratio of 0.38:0.64:0.97:1.26:1.00	*β*-configuration		
	GPP3-a		Rib, Fru, Gal, Glc in the ratio of 1.62:0.54:0.49:1.00	*α*-configuration		

8	GP-I	9.3×10^4^	Glc, Gal, Man, Rha, Ara in the ratio of 5.3: 4.2: 3.0: 0.7: 0.8		Anticancer	[43]

9	GP-B1	7.9×10^4^	Gal, Ara, Man, Rha, Xyl, Glc, GalA, GlcA in the ratio of 3.5:3.2: 0.6:0.9:0.3:0.5:0.6:0.4		Antitumor	[42]
	GP-C1	1.26×10^5^	Gal, Ara, Man, Rha, Glc, GlcA in the ratio of 2.1:1.0:0.3:0.5:0.4:0.9			

10	GPP2-s1	1.12×10^4^		C-6 position and C-2 position	Antitumor	[19]

11	GPP1-a	8.92×10^4^	Ara, Gal, Glc in the ratio of 0.18:0.72:1.00	Backbone composed of (1→4)-*α*-_D_- Glc*p*, with braches attached to O-6 of some residues. Braches composed of (1→6)-*α*-_D_-Glc*p*, (1→3)-*β*-_D_-Gal*p*, (1→6)-*α*-_D_-Gal*p*, and terminated with (1→)-*β*-_D_-Gal*p *and (1→)-*β*-_L_-Ara*f. α*-configuration.	Anti-fatigue activity	[22]
	GPP2-b	1.975×10^5^	Ara, Rib, Xyl, Gal, Glc in the ratio of 0.38:0.64:0.97:1.26:1.00	*α*-configuration		
	GPP3-a	2.536×10^5^	Rib, Fru, Gal, Glc in the ratio of 1.62:0.54:0.49:1.00	*α*-configuration		

12	PSGP		Gal, Ara, Rha, GalA, Xyl, Man, GlcA in the ratio of 18.9: 10.5:7.7: 4.7:3.9:3.1:1.2		Immunomodulation	[61]

13	GPP-S	1.2×10^6^	Rha, Ara, Glc, Gal in the ratio of 1:3.72:19.49:7.82	Backbone composed of (1→4)-linked- Glc*p *and (1→6)-linked-Gal*p*, Braches composed of (1→4,6)-*α*-_D_-Glc*p* and terminated with (1→)-linked-Ara*f* residues	Antioxidant Anti-inflammatory	[18]

14	GM		Glc, Gal, Man, Fru in the ratio of 1.54:3.05:1.00:1.10		Antioxidant	[62]
	GMA	9.4×10^4^	Glc, Fru in the ratio of 11.45:1.00			
	GMB	1.2×10^5^	Glc, Gal, Man in the ratio of 1.30:1.31:1.00			
	GMC	7.2×10^4^	Glc, Gal, Man, Fru in the ratio of 1.00:2.17:1.25:1.02			

15	GPP	7.1×10^3^	Man, Glc, Gal, Ara, in the ratio of 1.00:77.33:4.81:1.83.	Backbone composed of (1→4)-*α*-_D_- Glc*p*, with braches attached to O-6 of some residues. Braches composed of (1→4, 6)-*α*-_D_-Glc*p*, and terminated with (1→) -*α*-_D_-Glc*p *residues	Antioxidant	[15]

16	GPM1	2.0×10^5^	Rha, Ara, Xyl, Man, Glc, Gal in the ratio of 1.78:1.99:1.00:1.11:6.00:6.89		Antioxidant	[38]
	GPM2	1.67×10^5^	Rha, Ara, Xyl, Man, Glc, Gal in the ratio of 3.23:7.70:1.00:2.29:2.88: 14.82			

17	GPI		Glc, GalA, Man, Ara, Rha, Gal, Xyl in the ratio of 6.81:7.19:13.19: 33.86:6.77:8.13:3.46	furan structure	Antioxidant	[59]

18	GPP	2.52×10^6^	Man, GlcA, Gal, Xyl, Rha	*α*-configuration	Antioxidant	[53]

## Abbreviations

Man: Mannose
Rha: Rhamnose
GlcA: Glucuronic acid
GalA: Galacturonic acid
Glc: Glucose
Gal: Galactose
Xyl: Xylose
Ara: Arabinose
Fuc: Fucose
Rib: Ribose
Fru: Fructose
HSQC: Heteronuclear singular quantum correlation
COSY: Correlation spectroscopy
HMBC: Heteronuclear multiple bond correlation
HOSC: Hydroxyl radical scavenging capacity
ORAC: Oxygen radical absorbance capacity
SOD: Superoxide dismutase
GSH-Px: Glutathione peroxidase
T-AOC: Total antioxidant capacity
GSH: Glutathione
CAT: Catalase
NF-*κ*B: Nuclear factor-kappaB
PI3K/Akt: Phosphatidylinositol-3-kinase/serine/threonine kinase
MAPK: Mitogen-activated protein kinase
Nrf2/HO-1: Nuclear factor-erythroid 2 related factor 2/ heme oxygenase-1.
